# A Novel Gene Signature Associated With “E2F Target” Pathway for Predicting the Prognosis of Prostate Cancer

**DOI:** 10.3389/fmolb.2022.838654

**Published:** 2022-04-13

**Authors:** Haoran Xia, Miaomiao Wang, Xiaonan Su, Zhengtong Lv, Qiuxia Yan, Xiaoxiao Guo, Ming Liu

**Affiliations:** ^1^ Department of Urology, Beijing Hospital, National Center of Gerontology, Institute of Geriatric Medicine, Chinese Academy of Medical Sciences, Beijing, China; ^2^ Graduate School of Peking Union Medical College and Chinese Academy of Medical Sciences, Beijing, China; ^3^ Department of Urology, Zoucheng People’s Hospital, Zoucheng, China; ^4^ Peking University Fifth School of Clinical Medicine, Beijing, China

**Keywords:** “E2F target” pathway, prostate cancer, gene signature, prognosis, immune infiltration, therapeutic resistance

## Abstract

**Background:** The effect of the adenoviral early region 2 binding factors (E2Fs) target pathway on prostate cancer is not clear. It is necessary to establish an E2F target-related gene signature to predict prognosis and facilitate clinical decision-making.

**Methods:** An E2F target-related gene signature was established by univariate and LASSO Cox regression analyses, and its predictive ability was verified in multiple cohorts. Moreover, the enrichment pathway, immune microenvironment, and drug sensitivity of the activated E2F target pathway were also explored.

**Results:** The E2F target-related gene signature consisted of *MXD3*, *PLK1*, *EPHA10*, and *KIF4A*. The patients with high-risk scores showed poor prognosis, therapeutic resistance, and immunosuppression, along with abnormal growth characteristics of cells. Tinib drugs showed high sensitivity to the expression of *MXD3* and *EPHA10* genes.

**Conclusion:** Our research established an E2F target-related signature for predicting the prognosis of prostate cancer. This study provides insights into formulating individualized detection and treatment as well as provides a theoretical basis for future research.

## Introduction

Prostate cancer is the second most common male malignant neoplasia and the fifth leading cause of cancer death in men worldwide. With an increase in the aging population, 2.3 million new cases of prostate cancer and 740,000 deaths are expected to occur globally in the next 20 years. Despite a decline in global incidence rates, the incidence in China shows an annual increase of 2.6% (Culp et al., 2020). Early diagnosis plays a key role in the prognosis of prostate cancer. To further improve patient outcomes, new molecular markers need to be identified to allow a more reliable diagnosis and prognosis.

The adenoviral early region 2 binding factors (E2Fs) of the transcription factor family are critical regulators of cell cycle progression (Sun et al., 2007). In response to mutation or phosphorylation, RB1 is inactivated, causing E2Fs to detach from the E2F–RB1 complex to bind with certain promoters of the E2F target genes (Van den Heuvel and Dyson, 2008; Hallstrom and Nevins, 2009). The high expression of the E2F target gene plays a pivotal role in tumorigenesis and is related to poor prognosis in many tumors, including neuroblastoma (Molenaar et al., 2012), breast cancer (Oshi et al., 2021), high-grade serous ovarian cancer (Dahl et al., 2019), and prostate cancer (Wang et al., 2021).

In this study, we aimed to find a novel prognosis gene signature to guide further clinical decision-making for patients with prostate cancer. Briefly, the effect of the E2F target pathway on the poor prognosis of prostate cancer was determined by single-sample gene set enrichment analysis (ssGSEA), and then the prognostic gene set related to the E2F target pathway was established by weighted gene co-expression network analysis (WGCNA) and differentially expressed gene (DEG) analysis. Using a cohort from The Cancer Genome Atlas (TCGA) database, a gene signature was obtained by univariate and least absolute shrinkage and selection operator (LASSO) Cox regression analyses, and the risk value of the E2F target pathway was calculated. Then, we verified the enrichment of the E2F target pathway and the worse prognosis in the high-risk group in two separate cohorts from the International Cancer Genome Consortium (ICGC) and Gene Expression Omnibus (GEO) databases. In addition, the study explored functional differences among different risk groups. Our developed gene signature could facilitate early screening, predict prognosis, and provide patients with more individualized treatments.

## Methods

### Data Preparation and Processing

In total, 1,145 patients with prostate adenocarcinoma (PRAD) from TCGA, ICGC, and GEO databases were enrolled in this study. Of these, the data of 540 patients with PRAD from TCGA database were downloaded as the training cohort (https://portal.gdc.cancer.gov/). Two test cohorts were used, namely, test cohort I consisting of 357 patients with PRAD from the ICGC database (https://dcc.icgc.org/projects/PRAD-CA) and test cohort II consisting of 248 patients with PRAD from the GSE116918 database (radical radiotherapy with ADT) (https://www.ncbi.nlm.nih.gov/geo/query/acc.cgi?acc=GSE116918). All downloaded data included fragments per kilobase of sequences per million mapped reads (FPKM)-normalized RNA sequencing (RNA-seq) data, clinical characteristics annotation, and follow-up information such as biochemical recurrence (BCR), metastasis (Met), and overall survival (OS). Before further analysis, all RNA-seq data included were log2-transformed and normalized by the R package “sva.”

### Construction of E2F Target Signature

First, based on the HALLMARKS gene set from the Molecular Signatures Database (MSigDB) (http://www.gsea-msigdb.org/gsea/msigdb/search.jsp), ssGSEA was calculated using the R package “GSVA” in the training set (Lee et al., 2008). The R package “WGCNA” was used to perform WGCNA by using TCGA database mRNA matrix (Langfelder and Horvath, 2008). An adjacency matrix was constructed to describe the correlation strength between the nodes and then transformed to a topological overlap matrix (TOM). Next, hierarchical clustering was performed to identify the modules by setting the minimum number of genes in each module to 60. After merging similar modules, the module genes with the highest correlation to E2F target scores in ssGSEA results were identified. These genes were then combined with the E2F target gene set in HALLMARKS to obtain a new gene set, denoted as gene set A.

Simultaneously, Kaplan–Meier survival and log-rank tests (R package “survival”) were performed using gene set A and BCR information to screen genes associated with prognosis—which were labeled as gene set B. DEGs between TCGA-PRAD tumors and normal tissues were obtained by using R package “limma” (false discovery rate [FDR] < 0.05, log|fold change [FC]|> 1) (Ritchie et al., 2015). E2F target-related candidate prognostic genes were obtained by intersecting DEGs with gene set B. Furthermore, these genes were enrolled for inclusion in LASSO Cox regression (R package “glmnet”) (Tibshirani, 1997). Finally, the remaining genes after 10,000 contractions were selected, and their coefficients were recorded to obtain an E2F target-related gene signature.

### Statistical and Bioinformatics Analyses

All analyses and graphs were executed using R v4.0.3 (http://www.r-project.org) and GraphPad v8.0.3 (https://www.graphpad.com/). The Pearson correlation test was used to calculate the established inter-gene correlations in the gene signature. After the E2F target-related risk score was calculated based on the signature, the training set (TCGA cohort) and the two test sets (cohorts from ICGC and GEO) were divided into high- and low-risk groups according to the median risk score in the training set. Differences between the groups were calculated using *t*-tests and chi-square tests. Scatter maps and heat maps were used to visualize the survival distribution and gene expression patterns of patients in different groups (R packages “ggplot2” and “scales”). Furthermore, the dimension of high-latitude data was reduced by using *t*-distributed stochastic neighbor embedding (tSNE) and principal components analysis (PCA) of the R package “Rtsne” to test whether the gene signature could divide patients into different groups (Reich et al., 2008; van der Maaten and Hinton, 2008). The prognostic ability of the gene signature was reflected by the Kaplan–Meier survival analysis, receiver operating characteristic (ROC) curve, and time-dependent ROC (T-ROC) curve (R packages “survival” and “timeROC”). The multivariate Cox stepwise regression models of the R package “survival” were used to test whether the gene signature was an independent predictor of prognosis.

The function between groups was analyzed by GSEA v4.1.0. For this algorithmic analysis, it was verified whether the E2F target pathway was activated in different groups. In addition, GSEA based on Gene Ontology (GO) and Kyoto Encyclopedia of Genes and Genomes (KEGG) databases was used to study functional differences according to the DEGs in different groups (R package “GOplot” and “limma”) (Subramanian et al., 2005).

The immune cells were divided into different types based on previous studies. Meanwhile, the differences of immune infiltration were explored by ssGSEA (immune gene set from MSigDB) and the CIBERSORT algorithm (https://cibersortx.stanford.edu/), as well as the immunophenoscore (IPS) and immunophenotyping (Newman et al., 2015; Charoentong et al., 2017; Thorsson et al., 2018). Finally, gene correlation analysis based on the CellMiner database (https://discover.nci.nih.gov/cellminer/home.do) explored the sensitivity between the genes and drugs to screen for new medicines and therapeutic targets (Pommier et al., 2012).

## Results

### E2F Target Pathway Identified as a Prognostic Risk Factor

**FIGURE 1:**
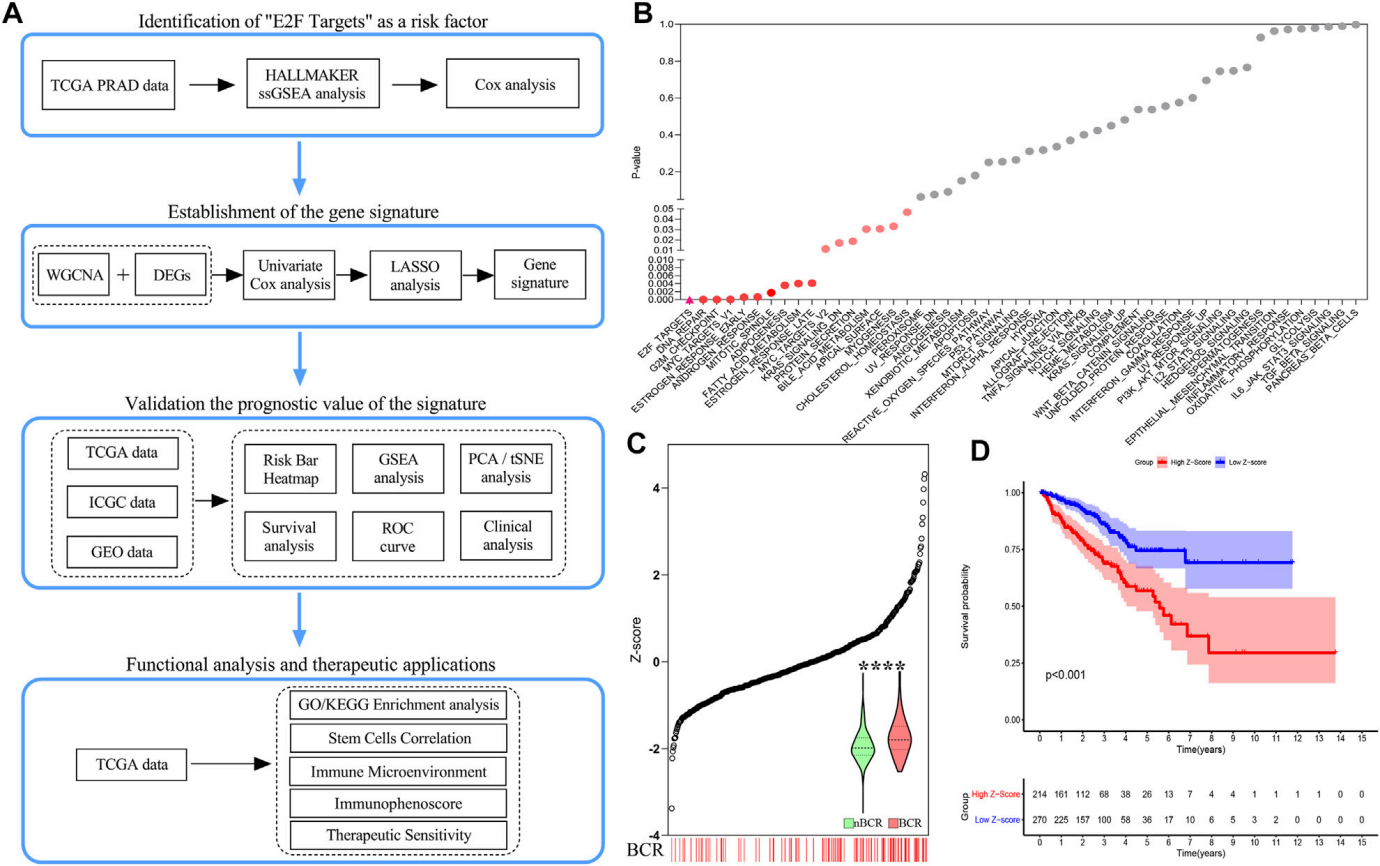
Schematic diagram and identification of risk factors for the “E2F target” pathway. **(A)** Research design and process diagram. **(B)** Univariate Cox regression analysis revealed a significant correlation between the “E2F target” pathway and poor prognosis. **(C)** The number of BCR patients increased significantly with increasing z-score. **(D)** The prognosis of the high z-score group was worse than that of the low z-score group by Kaplan–Meier analysis.

The overall process is shown by a schematic diagram in Figure 1A. In the first step, ssGSEA scores of 50 pathways in each sample were included in univariate Cox regression analysis. For the BCR of the training cohort, the E2F target pathway had the greatest statistical significance (*p* < 0.0001, Figure 1B). The E2F target pathway score was z-score-transformed and divided into high- and low-z-score groups according to their median value. The incidence of BCR in the high-z-score group was significantly higher than that of the low-group (*p* < 0.0001, Figure 1C). Moreover, the Kaplan–Meier survival curves revealed a worse prognosis in the high-z-score group (*p* < 0.001, Figure 1D). The details of the training cohort are presented in Supplementary Table S1.

### Candidate Gene Screening and Gene Signature Construction

**FIGURE 2:**
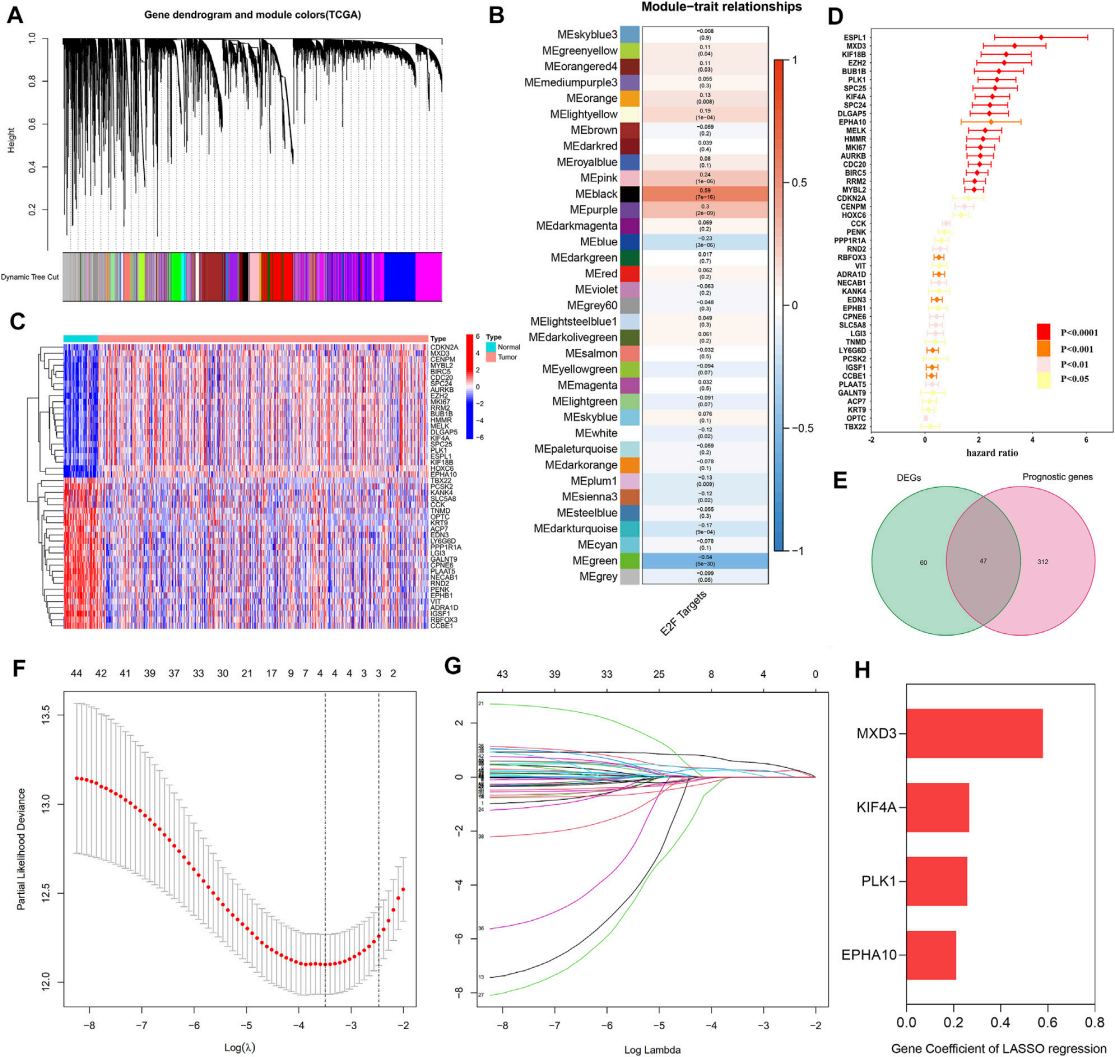
Establishment of the “E2F target” risk score. **(A–B)** WGCNA showed that the gene in MEblack had the highest correlation with the activation of the “E2F target” pathway. **(C)** The heat map showed the top part of DEGs between the prostate tumor and normal prostate tissue. **(D)** Prognostic genes with statistical significance were screened by univariate Cox regression analysis. **(E)** The Venn diagram showed 47 prognostic differential genes obtained after the intersection of DEGs and prognostic genes. **(F–G)** LASSO Cox regression was used to establish the signature; the best log(λ) value was -3.5, and 4 indicators remained. **(H)** The LASSO coefficients of the 4 genes in the signature.

A total of 35 gene modules were identified by WGCNA, among which the expression of the black module was most related to the increase in the E2F target z-score, with a total of 806 genes (Figures 2A,B and Supplementary Table S2). Then, 1,006 genes related to the E2F target pathway were obtained by combining the 806 genes with those of the E2F target pathway from MSigDB (Supplementary Table S2). After univariate Cox regression analysis, 359 E2F-related prognostic genes were screened out (Figure 2C and Supplementary Table S3). Concurrently, 107 DEGs were identified by differential analysis between cancer and normal prostate tissue (Figure 2D and Supplementary Table S3), and a total of 44 candidate “E2F target”-related prognostic genes were obtained by intersecting these DEGs with the previously screened genes (Figure 2E and Supplementary Table S3). Subsequently, the 44 candidate genes were further screened by a LASSO Cox regression model, optimized when the minimum λ value was 0.03035, with four genes remaining, including MAX dimerization protein 3 (MXD3), polo-like kinase 1 (PLK1), EPH receptor A10 (EPHA10), and kinesin family member 4A (KIF4A) (Figures 2F,G). Figure 2H and Supplementary Table S3 show the coefficients of these four genes, and the final risk score was calculated as follows:

Risk score = MXD3* 0.579083868008915 + PLK1* 0.258020225608399 + EPHA10* 0.211234188276561 + KIF4A * 0.266044684281992.

### High E2F Target-Related Risk Score Associated With Poor Prognosis in Training Cohort

**FIGURE 3:**
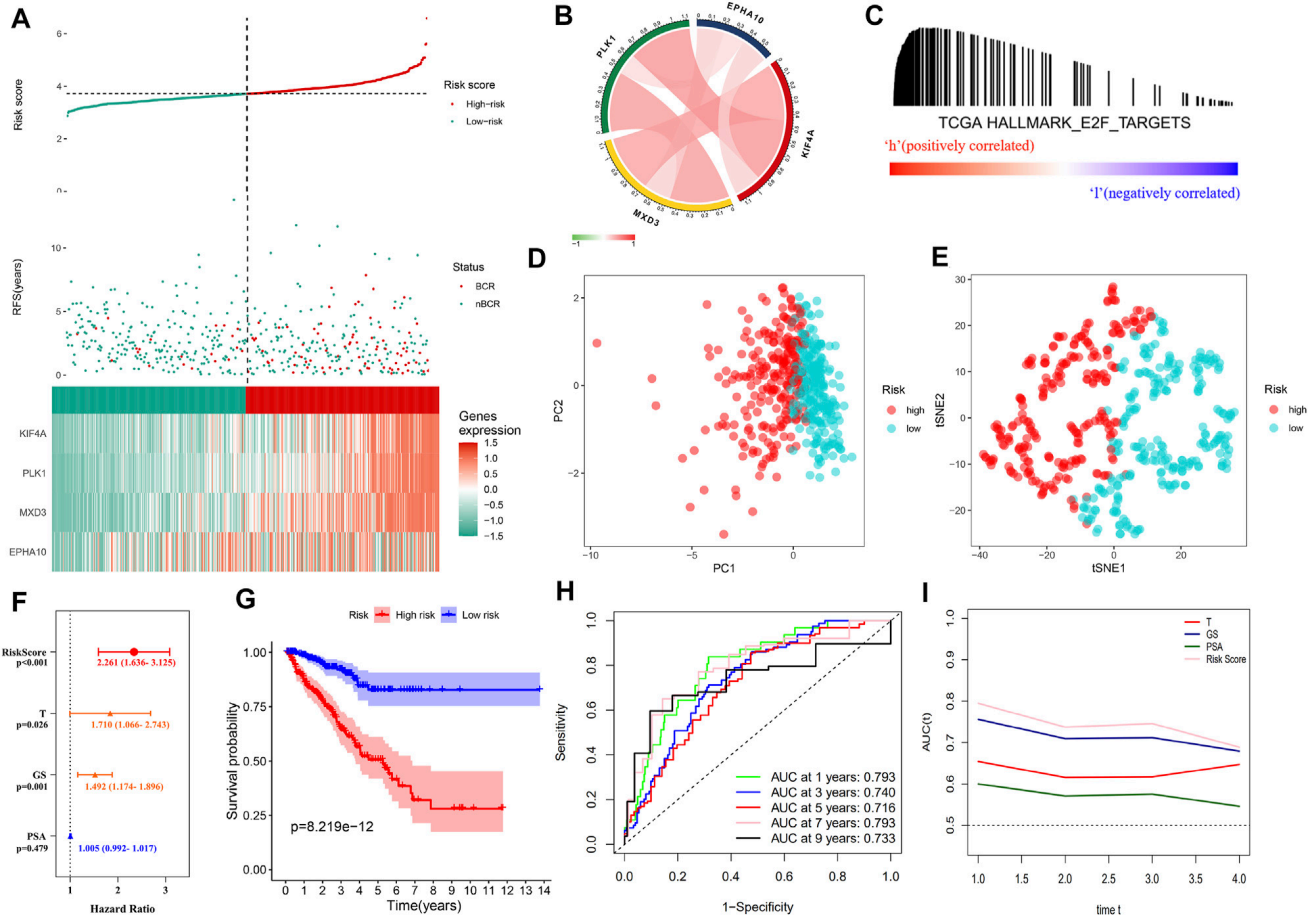
Distribution of the “E2F target” risk score in TCGA cohort. **(A)** Patient risk score–survival distribution map and gene expression heat map of “E2F target”-related genes. **(B)** Gene correlation heat map showing low correlation among genes. **(C)** GSEA proved that in the high-risk group, the “E2F Target” pathway was activated. **(D–E)** PCA and tSNE analysis indicated that the model could be used to distinguish between different risk groups. **(F)** The “E2F target” risk score was an independent prognostic factor for poor prognosis. **(G)** Kaplan–Meier survival analysis confirmed that patients with a high-risk had a worse prognosis. **(H)** The 10-year AUC determined by ROC analysis of the gene signature was relatively high, suggesting that the predictive ability of the signature was good. **(I)** Compared with the T stage, Gleason score, and PSA, the average AUC of the risk score was the highest, indicating that the predictive ability of the risk score was the best.

As the risk score increased, the gene expression of the signature rose, the number of patients with BCR in the training cohort increased significantly, and the BCR-free survival time decreased (Figure 3A). The genetic correlation analysis did not show excessive high correlations (Figure 3B and Supplementary Table S4). According to the median risk score of 3.713494217, the patients were divided into high- and low-risk groups. GSEA confirmed the enrichment of the E2F target pathway genes in the high-risk group (Figure 3C). PCA and tSNE analysis showed that patients in the high- and low-risk groups could be completely distinguished from each other (Figures 3D,E). Multivariate Cox regression analysis showed that the risk score was an independent predictor of prognosis and the strongest predictor of BCR along with clinical features (HR: 2.261, 95% CI: 1.636–3.125, *p* < 0.001, Figure 3F). The Kaplan–Meier survival curve showed that the BCR-free survival time in the high-risk group was significantly lower than that in the low-risk group (*p* < 0.0001, Figure 3G). Figure 3H shows that the area under the ROC curve (AUC) of the risk score in the training group within 9 years was all higher than 0.7, which indicates that the average predictive ability of the risk score was strong. The T-ROC curve revealed that the AUC of the risk score was always higher than that of other clinical features and tended to be stable over time (Figure 3I).

### Verification of the Prognostic Effect of E2F Target-Related Risk Score in the Test Cohort

**FIGURE 4:**
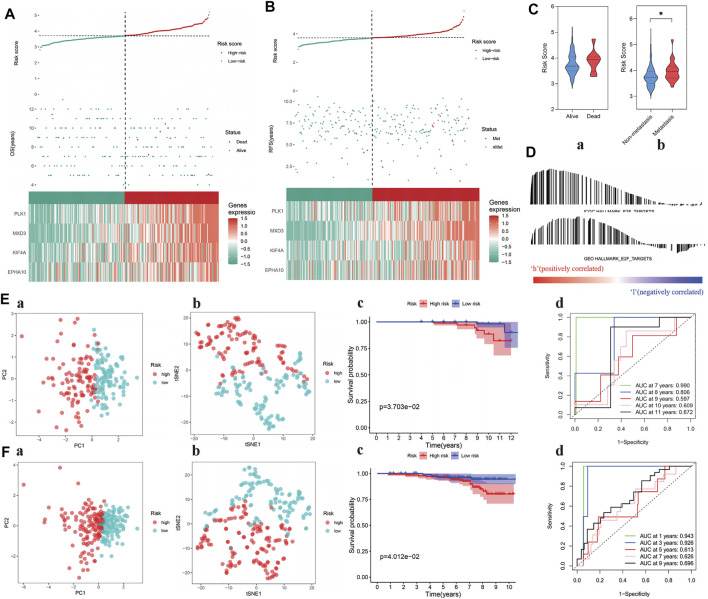
Verification of prognostic ability of “E2F target” risk score in ICGC and GEO cohort. **(A–B)** Patients’ risk score–survival distribution map and gene expression heat map of “E2F target”-related genes in ICGC (left) and GEO (right) cohorts. **(C)** The “E2F target” risk score of patients with poor prognosis increased in both ICGC (left) and GEO (right) cohorts. **(D)** GSEA proved that at the high-risk group, the “E2F target” pathway was activated in both cohorts. **(E–F)** The ability of the “E2F target” risk score to distinguish high-risk patients was verified by PCA and tSNE in ICGC (Ea, Eb) and GEO (Fa, Fb) cohorts, and the prognosis of the high-risk group was significantly worse than the low-risk group (Ec, Ed, Fc, and Fd).

The risk score of each sample was calculated in test cohorts I and II, which were divided into high- and low-risk groups according to the median risk score of the training cohort (i.e., 3.713494217). The four-gene signature showed the same expression pattern as the training cohort, and the distribution of deaths in test cohort I and metastatic patients in test cohort II increased with the risk score (Figures 4A–C). In addition, in the high-risk groups of the two cohorts, the E2F target pathway was shown to be activated (Figure 4D). In addition, the composition of the gene signature was verified to distinguish between patients at different risks and to predict poor prognosis within 10 years in the two cohorts (Figures 4E,F). The detailed risk information and gene expression of the training and test cohorts are represented in Supplementary Table S5, and the relevant information generated in the process of GSEA is shown in Supplementary Table S6.

### Correlation Between Gene Signature and Clinical Characteristics

**FIGURE 5:**
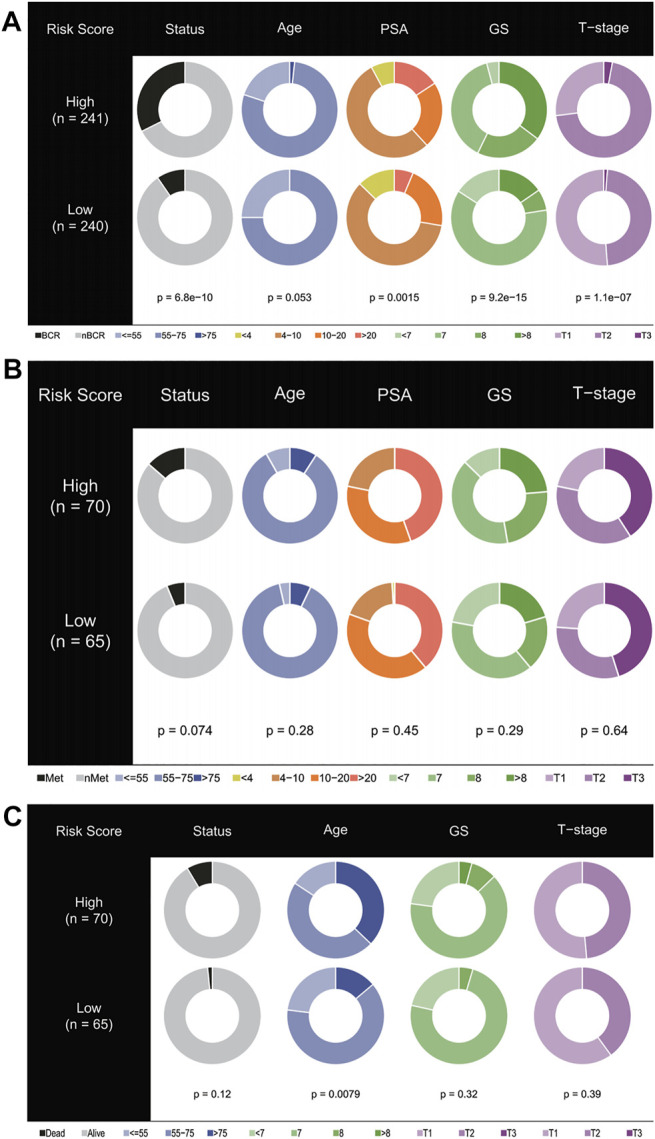
Stratified proportion of clinical characteristics of patients with prostate cancer in high- and low-risk groups. In the TCGA **(A)**, ICGC **(B)**, and GEO **(C)** cohorts, patients with higher PSA, GS, and T stage accounted for an increase in the high-risk group.

Pearson correlation analysis was used to study the distribution of patients among different groups with various clinical feature stratifications. As shown in Figure 5A, in the training cohort, the high-risk group was closely associated with higher PSA, Gleason score (GS), and T stages. Similarly, in test cohorts I and II, the PSA, GS, and T stages tended to increase in the high-risk group (Figures 5B,C). Unfortunately, this trend was not statistically significant. In this regard, we gave a cautious explanation. In test cohort I, as OS was the primary endpoint, a shortage of other endpoints arose, which thus affected the statistically significant results. In contrast, test cohort II received radiotherapy plus androgen deprivation therapy, which, from the perspective of the E2F target pathway, may have been more beneficial to patients, thus affecting OS and the statistically significant results.

### Functional Differences Among Different Risk Groups

**FIGURE 6:**
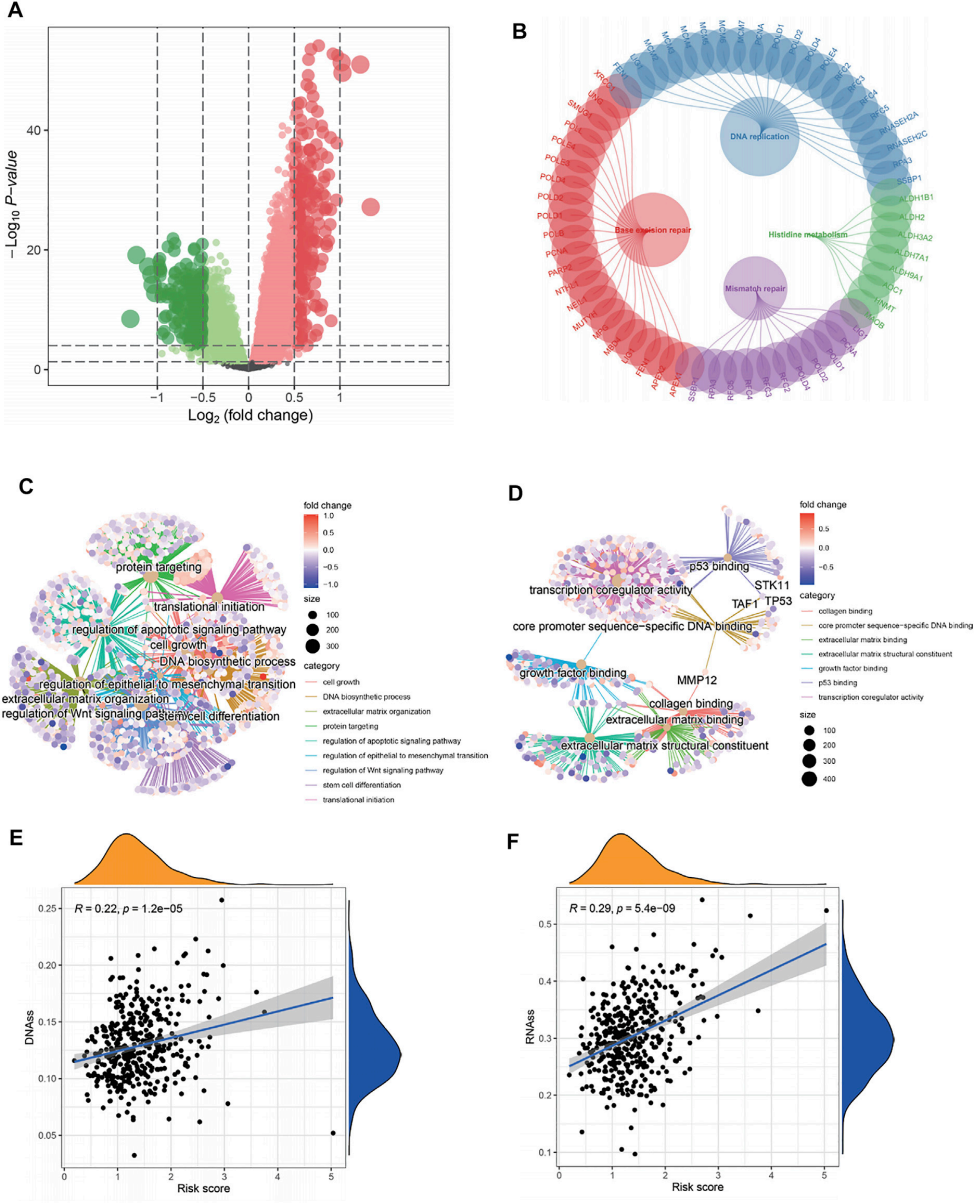
Functional analysis and correlation analysis of stem cell characteristics and risk score. **(A)** Volcano diagram of DEGs between high- and low-risk groups. **(B–D)** KEGG and GO enrichment analysis showed that DNA replication, mismatch repair, cell growth, extracellular matrix remodeling, and binding pathways were active in patients with a high-risk, as well as the p53 pathway, wnt pathway, and stem cell differentiation. On the contrary, histidine metabolism was inhibited. **(E–F)** The “E2F target” risk score was positively correlated with DNAs and RNAs.

The DEGs in the high- and low-risk groups were calculated using the “limma” algorithm. Figure 6A shows all the up- and downregulated DEGs screened by using *p* < 0.05 as a threshold. The functional enrichment analysis of DEGs was performed, and the results of KEGG analysis showed that DNA replication, base excision repair, and mismatch repair were active in the high-risk group. Surprisingly, histidine metabolism was suppressed in this group (Figure 6B). As shown in Figures 6C,D and Supplementary Table S7, the GO analysis showed that in the high-risk group, the activation of the E2F target pathway participated in the binding of many factors and proteins and may regulate cell growth, transcription, apoptosis, and other metabolic activities by interfering with binding processes. The study also found the regulation of the Wnt and p53 signaling pathways in the high-risk group, suggesting that these pathways may be abnormally activated. In addition, the study found that the activation of the E2F target pathway was also related to remodeling of the extracellular matrix, which may enhance the invasive ability of tumor cells, promote cancer metastasis, and lead to poor prognosis. Moreover, the correlation between the risk scores and the characteristics of tumor stem cells was studied. A significant positive correlation was found between the risk score and stem cell characteristics (Figures 6E,F). All relevant information to support these results is presented in Supplementary Table S7.

### Immune Microenvironment and Therapeutic Sensitivity

**FIGURE 7:**
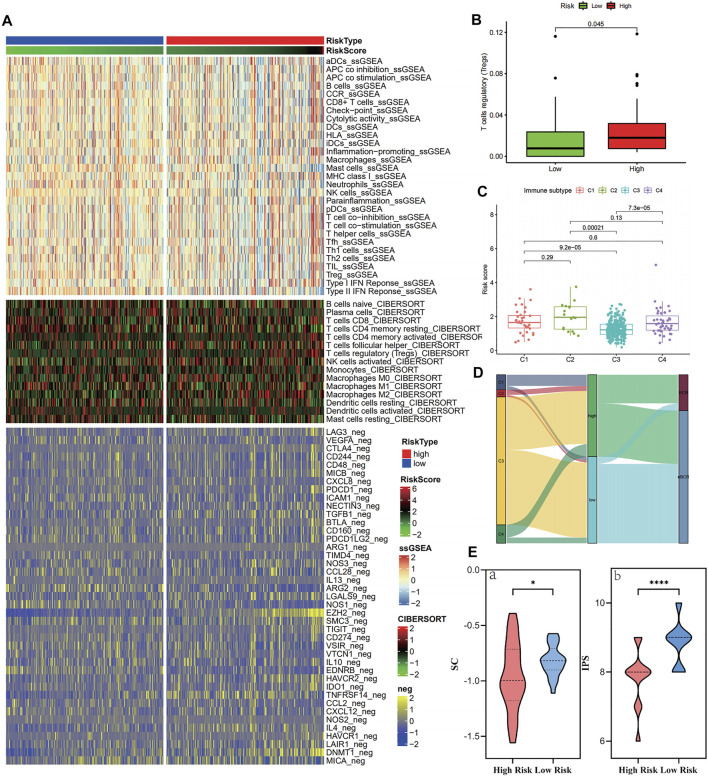
Immune cell infiltration in the high- and low-risk groups in the training cohort. **(A–B)** Tregs and negative immunoregulatory genes were highly expressed in the high-risk score group. **(C–D)** Immunotyping results showed that C1, C2, and C4 types were increased in the high-risk group. **(E)** The IPS algorithm showed a significant increase in SC (a) and CP (b) in the high-risk group.

Figure 7A and Supplementary Table S8 show the immune infiltration of high- and low-risk groups using different algorithms. From the ssGSEA and CIBERSORT thermograms, the immune infiltration of patients in the high-risk group was more complex with both immune activation and immunosuppression and varying degrees. However, it is worth noting that both the algorithms showed significant infiltration of T regulatory cells in the high-risk group (Figure 7B). Concomitantly, negative immune regulatory genes, such as *EZH2*, *HAVCR1/2*, and *DNMT1* were also observed to be significantly expressed in the high-risk groups. When focusing on the immune subtype, it was found that the C3 type with good prognosis was concentrated in patients with low-risk scores, while the higher the risk score, the more the C1, C2, and C4 types were linked to poor prognosis (Figures 7C,D). Continuously, the IPS algorithm was used to calculate the immunophenotype of the samples with the top 10 and the last 10 risk scores, which once again verified the high expression of immune suppressor cells (SCs) and a decrease in the IPS in the high-risk group (Figure 7E and Supplementary Figure S1).

**FIGURE 8:**
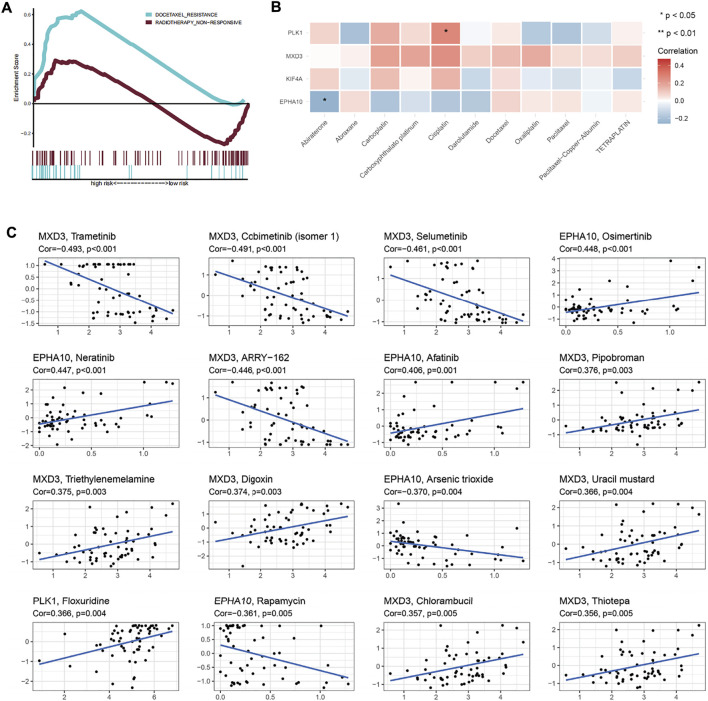
Therapeutic response and drug sensitivity analysis. **(A)** Patients in the high-risk group showed significant docetaxel resistance. **(B)** Sensitivity analysis of genes in “E2F target”-related signature and common drugs of prostate cancer. **(C)** Drug sensitivity analysis showed that the top 16 drugs in the CellMiner database, which were sensitive to genes in “E2F target”-related signature.

GSEA and sensitivity analysis of commonly used drugs reflected the resistance of high expression of different genes to treatment (Figures 8A,B and Supplementary Table S9). *MXD3* had certain sensitivity to commonly used drugs, although not that strong. In addition, sensitive drugs were screened using the CellMiner database (Supplementary Table S9), and the results showed that -tinib drugs were more sensitive to *MXD3* and *EPHA10*, suggesting that they may become potential new therapeutic targets.

## Discussion

The progression of prostate cancer is interrelated with multiple genes. Certain tumor molecular markers can predict the prognosis of patients for more precise personalized treatment plans in patients with cancer. Retinoblastoma tumor suppressor protein (RB) is a pivotal regulator of the cell cycle and functionally inactive in most cancers, including prostate cancer (Classon and Harlow, 2002; Jarrard et al., 2002). A previous study showed that RB plays a tumor suppressor function in prostate cancer (Bookstein et al., 1990) and plays a key role in the cell cycle regulation by regulating the adenoviral early region 2 binding factors (E2Fs) of transcription factor family (Fischer and Müller, 2017; Wu and Wu, 2021). The E2F family of transcription factors (E2Fs) play an important role in cell cycle regulation; E2F1, E2F3, and E2F7 were reportedly involved in the G1-S transition procession of the cell cycle (Xie et al., 2021). E2F transcription factors not only regulate the expression of target genes but also ensure that target genes are mainly transcribed in a cell cycle-dependent manner (Fang et al., 2020). Therefore, E2F transcription factor abnormalities play a role in tumorigenesis. Mutations in RB are inactivated in prostate cancer, leading to RB–E2F complex dissociation, followed by free E2F binding to the promoter of certain E2F target genes, in turn controlling the progression of tumorigenesis. Various E2F target genes are related to one another and are expected to form a gene signature to predict patient prognosis (Kent and Leone, 2019). Therefore, E2F target genes play an important role in cancer development.

In this study, bioinformatics analysis was used to find four E2F target-related genes as a novel prognosis gene signature to guide further clinical decision-making. The four gene signatures (*MDX3*, *PLK1*, *EPHA10*, and *KIF4A*) showed a strong correlation with prostate cancer prognosis in cases selected from TCGA database. These genes may predict the prognosis of prostate cancer more accurately than existing signatures. We also verified the enrichment of the E2F target pathway and the worse prognosis in high-risk groups from two separate cohorts of the ICGC and GEO databases. In addition, exploring the functional differences among different risk groups showed that in high-risk groups, the binding process of a variety of proteins and molecules was abnormally activated, and the properties of stem cells increased. Simultaneously, T regulatory cells and immunosuppressive genes were highly expressed. These may lead to abnormal proliferation, apoptosis, extracellular matrix remodeling, and immune escape of tumor cells. It is worth noting that severe inhibition of histidine metabolism was observed in the high-risk group. Some studies have found that accelerating histidine metabolism may improve the therapeutic effect of anticancer drugs (Kanarek et al., 2018). Therefore, the activation of the E2F target pathway may cause resistance to the treatment by inhibiting the histidine metabolism. Accordingly, our drug analysis demonstrated the low sensitivity of drugs and hinted at the potential of *MXD3* and *EPHA10* as therapeutic targets.

Recent studies show that PLK1 and KIF4A as biomarkers have a high prognosis value in patients with prostate cancer (Gao et al., 2018; Cao et al., 2020; Liu et al., 2020; Wu et al., 2020; Das et al., 2021). PLK1 has been proven to be a potent and promising target for prostate cancer treatment (Mao et al., 2018; Shin et al., 2019). MXD3 is a transcription target of E2F1 (Yun et al., 2007) and belongs to the MYC/MAX/MAD network, which can compete with MYC to regulate the cell cycle and proliferation (Ayer et al., 1993; Grandori et al., 2000). MXD3 has been shown to predict poor prognosis in clear cell renal cell carcinoma (Zhang et al., 2021) and hepatocellular carcinoma (Xu et al., 2019), as well as having been indicated as a new molecular targeted site to treat neuroblastoma (Yoshida et al., 2020). *PLK1* (Iliaki et al., 2021), *EPHK10*, and *KIF4A* also play important roles as prognostic indicators or as targeted therapy sites for the progression of multiple tumors, such as pancreatic cancer (Zhu et al., 2021), esophageal squamous cell carcinoma (Li et al., 2021), bladder cancer (Zheng et al., 2021), and lung cancer (Kirienko et al., 2021). Furthermore, EPHA10 has already been approved as a potential therapeutic target of prostate cancer (Nagano et al., 2014), and high *EPHA10* expression correlated with lymph node metastasis of breast cancer (Nagano et al., 2013)—the type of malignant behavior that usually predicts a poor prognosis.

Our study is not without limitations. First, although the study used advanced bioanalysis algorithms for analysis, the study was based on the analysis of mRNA levels, and no experiments were performed to validate the effect of gene expression on phenotype. Second, due to the complexity of prostate cancer treatment and defects in the use of public databases, different patients in the same cohort may have received different treatments, which may have a certain impact on gene expression and cause bias in the results. Moreover, because prostate cancer is inert cancer, the study chose different prognostic endpoints in different cohorts, which may affect the comparison of risk values. To address these problems, it is necessary to establish prospective cohorts and perform in-depth experiments to elucidate potential pathways and mechanisms.

Nevertheless, this research introduces many innovations and commendable points. This study established the first E2F target-related gene signature to predict the prognosis of prostate cancer. It used cohorts from TCGA and ICGC databases with the same treatments, minimizing cohort heterogeneity to ensure the reliability of the analysis results. In addition, a cohort of radiotherapy plus androgen deprivation therapy from the GEO database was used to further verify the ability of the gene signature to predict the prognosis of patients at different stages of treatment. Consequently, further functional analysis and immune microenvironment analysis provided a preliminary explanation for the tumor-promoting effect of the E2F target pathway and revealed potential therapeutic target genes and sensitive drugs.

## Conclusion

This research established the first gene signature related to the E2F target pathway to predict the prognosis of prostate cancer. Furthermore, possible explanations for how the activation of the E2F target pathway results in the occurrence and development of prostate cancer have been provided. The guidance offered by this study can be useful for the individualized detection and treatment of patients with prostate cancer and lay a theoretical basis for further research into the therapeutic potential of the E2F target pathway.

## Data Availability

The original contributions presented in the study are included in the article/Supplementary Material, further inquiries can be directed to the corresponding authors.

## Supplementary Material

Supplementary Figure S1 Pie chart displayed the z-score of MHC, EC, CP, and SC of IPS algorithm in the top 10 and the last 10 “E2F target” risk score of patients.

## Glossary

AUC: area under the curve
BCR: biochemical recurrence
DEGs: differentially expressed genes
E2F: adenoviral early region 2 binding factor
EPHA10: EPH receptor A10
FC: fold change
FDR: false discovery rate
FPKM: fragments per kilobase million
GEO: Gene Expression Omnibus
GO: Gene Ontology
GS: Gleason score
ICGC: International Cancer Genome Consortium
IPS: immunophenoscore
KEGG: Kyoto Encyclopedia of Genes and Genomes
KIF4A: kinesin family member 4A
LASSO: least absolute shrinkage and selection operator
Met: metastasis
MSigDB: Molecular Signatures Database
MXD3: MAX dimerization protein 3
OS: overall survival
PCA: principal components analysis
PLK1: polo-like kinase 1
PRAD: prostate adenocarcinoma
RB: retinoblastoma tumor suppressor protein
RNA-seq: RNA-sequencing
ROC: receiver operating characteristic
SCs: suppressor cells
ssGSEA: single-sample gene set enrichment analysis
TCGA: The Cancer Genome Atlas
TOM: topological overlap matrix
T-ROC: time-dependent ROC
tSNE: t-distributed stochastic neighbor embedding
WGCNA: weighted gene co-expression network analysis.
