# Supplementary figures and images for: A Novel Gene Signature Associated With “E2F Target” Pathway for Predicting the Prognosis of Prostate Cancer
Source: Front Mol Biosci. 2022 Apr 13;9:838654. doi: 10.3389/fmolb.2022.838654 (PMC9045651; doi:10.3389/fmolb.2022.838654)

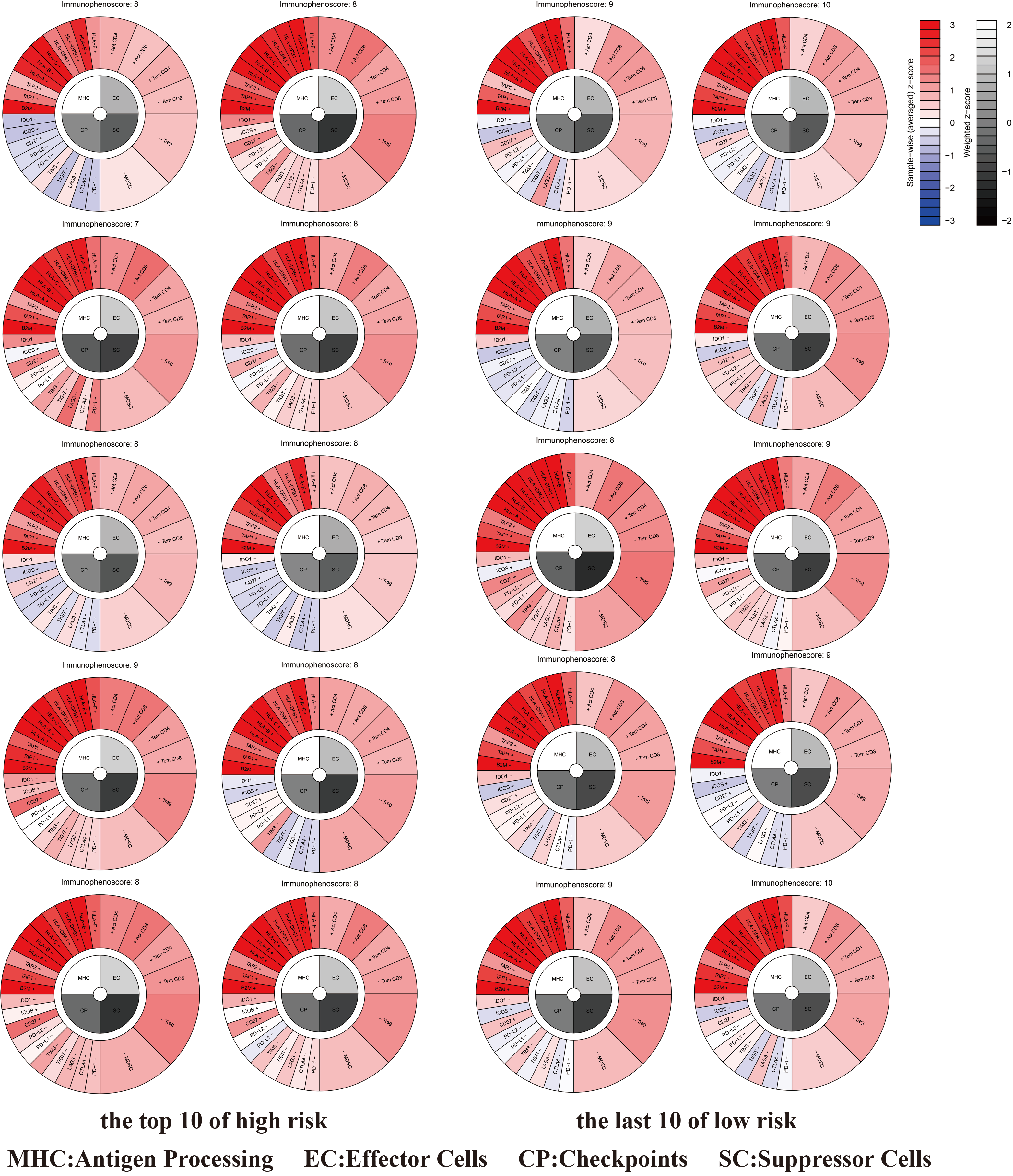

Supplement: Supplementary file 6 [file Image1.TIF]
